# Insulin resistance and endocrine-metabolic abnormalities in polycystic ovarian syndrome: Comparison between obese and non-obese PCOS patients

**Published:** 2016-04

**Authors:** Parvin Layegh, Zohreh Mousavi, Donya Farrokh Tehrani, Seyed Mohammad Reza Parizadeh, Mohammad Khajedaluee

**Affiliations:** 1 *Endocrine Research Center, Imam Reza Hospital, Faculty of Medicine, Mashhad University of Medical Sciences, Mashhad, Iran.*; 2 *Vascular and Endovascular Research Center, Imam Reza Hospital Faculty of Medicine, Mashhad University of Medical Sciences, Mashhad, Iran. *; 3 *Biochemistry and Nutrition Research Center, Faculty of Medicine, Mashhad University of Medical Sciences, Mashhad, Iran.*; 4 *Department of Community Medicine, Mashhad University of Medical Sciences, Mashhad, *Iran.

**Keywords:** *Polycystic ovary syndrome (PCOS)*, *Insulin resistance*, *Obese*, *Non-obese*

## Abstract

**Background:**

Insulin resistance has an important role in pathophysiology of polycystic ovarian syndrome (PCOS). Yet there are certain controversies regarding the presence of insulin resistance in non-obese patients.

**Objective:**

The aim was to compare the insulin resistance and various endocrine and metabolic abnormalities in obese and non-obese PCOS women.

**Materials and Methods:**

In this cross-sectional study which was performed from 2007-2010, 115 PCOS patients, aged 16-45 years were enrolled. Seventy patients were obese (BMI ≥25) and 45 patients were non-obese (BMI <25). Presence of insulin resistance and endocrine-metabolic abnormalities were compared between two groups. Collected data were analyzed with SPSS version 16.0 and p<0.05 was considered as statistically significant.

**Results:**

There was no significant difference in presence of insulin resistance (HOMA-IR >2.3) between two groups (p=0.357). Waist circumference (p<0.001), waist/hip ratio (p<0.001), systolic (p<0.001) and diastolic (p<0.001) blood pressures, fasting blood sugar (p=0.003) and insulin (p=0.011), HOMA-IR (p=0.004), total cholesterol (p=0.001) and triglyceride (p<0.001) were all significantly higher in obese PCOS patients. There was no significant difference in total testosterone (p=0.634) and androstenedione (p=0.736) between groups whereas Dehydroepiandrotendione sulfate (DHEAS) was significantly higher in non-obese PCOS women (p=0.018). There was no case of fatty liver and metabolic syndrome in non-obese patients, whereas they were seen in 31.3% and 39.4% of obese PCOS women, respectively.

**Conclusion:**

Our study showed that metabolic abnormalities are more prevalent in obese PCOS women, but adrenal axis activity that is reflected in higher levels of DHEAS was more commonly pronounced in our non-obese PCOS patients.

## Introduction

Polycystic ovary syndrome (PCOS) is a common endocrine disorder in reproductive-aged women. Hirsutism, seborrehea and acne due to hyperandrogenemia and menstrual irregularity and infertility are among most common complaints of these patients. Women with PCOS have various degrees of insulin resistance and disturbances in insulin secretion and function have an important role in pathophysiology of this disorder. PCOS patients have a collection of cardiovascular risk factors that are referred to as metabolic or X-syndrome (1). Although obesity is a common finding in PCOS subjects, it is not essential for making this diagnosis and about half of these patients are virtually not obese. 

On the other hand, insulin resistance is common in PCOS patients even in non-obese cases (2). Despite the generally accepted fact that obese PCOS subjects have insulin resistance and insulin sensitivity in these patients is less than obese non-PCOS women, the findings in non-obese PCOS patients are controversial. Several studies have not reported insulin resistance in non-obese PCOS subjects, whereas others have reported this disorder in such patients (3-8). Differences in genetics, ethnicity, nutrition and lifestyle have a certain role in findings of various studies. 

So, with respect to importance of insulin resistance in occurrence of endocrine and metabolic disorders in PCOS patients, controversies on presence of insulin resistance in non-obese subjects and also the role of genetics and ethnicity in the observed differences among different studies, we designed this study to compare the existence of insulin resistance and its endocrine-metabolic consequences in obese and non-obese PCOS patients.

## Materials and methods

This cross-sectional study was performed during 2007-2010 in Khorasan Province in north-east of Iran. The study was approved by the Ethics Committee of Mashhad University of Medical Sciences (MUMS) and a written informed consent was obtained from each participant prior to the study entrance. 

115 PCOS patients, age from 16-45 years were enrolled. We used the Rotterdam 2003 criteria for selection of the patients as 2 out of 3 criteria (9): 1. oligo- and/or anovulation 2. clinical and/or biochemical signs of hyperandrogenism 3. polycystic ovaries and exclusion of other etiologies such as CAH, Cushing syndrome and androgen-secreting tumors. 

Patients with hyperprolactinemia, non-classic CAH, Cushing syndrome, acromegaly, hypothyroidism, ovarian failure, adrenal and ovarian neoplasm and simple obesity were excluded based on history, clinical examination and appropriate laboratory tests wherever required. Patients having used any type of medication in previous three months and patients with known diabetes mellitus were also excluded. 

A specifically-designed questionnaire including demographic information, chief complaints, history of menstrual cycles, duration of disorder, family history of disease, presence of hirsutism, acne and alopecia was filled for each patient at study entrance. Venous blood sample was obtained after 12 hr fasting at 8:00 AM and glucose, insulin, lipid profile, total testosterone, androstenedion, LH, FSH, 17-OHP, hsCRP, estradiol, prolactine and TSH were measured. Glucose and insulin levels were also measured 2 hr after 75 gr glucose load. The samples were obtained in follicular phase of menstrual cycle (spontaneously or induced with medroxyprogestrone). Ultrasound study of liver (for signs of fatty liver) and ovaries was done by single radiologist for all patients. Mean volume of both ovaries were calculated for each patient. 

HOMA-IR was calculated as: fasting insulin (µu/ml)×fasting glucose (mmol/lit)/22.5. We used NECP-ATP III (2005) for defining metabolic syndrome (3 of 5 below criteria: fasting blood suger≥100 mg/dl, high density lipoprotein <50 mg/dl, triglyceride ≥150 mg/dl, waist circumference ≥88 cm and systolic blood pressure ≥130 and/or diastolic pressure ≥85). Scoring of acne was done as follows: 0= no acne, 1= comedone without inflammatory papules or pustules, 2= multiple inflammatory lesions but rarely cysts, 3= multiple inflammatory papules or pustules with cysts. Severity of alopecia was measured based on Ludwig score (A-C). Hirsutism was rated by the Ferriman-Gallwey scoring system (9 body areas each rated from 0-4). 

**Statistical analysis**

The collected data were analyzed with SPSS (Statistical Package for Social Sciences, Version 16.0, SPSS Inc, Chicago, Illinois, USA) and p<0.05 was considered as statistically significant. We used Student’s t-test and Mann-Whitney U test for continuous data based on their distribution. ^2^and Fisher’s exact test was used for nominal data whenever indicated.

## Results

In total 115 PCOS patients were studied; 70 patients with BMI ≥25 (obese) and 45 with BMI <25 (non-obese). Mean age of participants was 24.51±5.44 years (16-39 yrs). Mean disease duration was 8.35±5.19 yrs (2-25 yrs). Positive family history of PCOS was seen in 56.1% of non-obese and 57.1% of obese subjects (p=0.915). No significant difference in various degrees of acne was observed between the two groups (p=0.229). Chief complaints comparison of obese and non-obese patients is presented in table I.

Based on Ludwig score none of patients in two groups had significant alopecia. Fatty liver based on ultrasound study was diagnosed in 31.3% of obese but none of non-obese patients (p<0.001). Metabolic syndrome was seen in 39.4% of obese patients but none of non-obese ones (p<0.001). Abnormal fasting glucose (≥100 mg/dl) was seen in 36.9% of obese and 10.5% of non-obese PCOS patients (p=0.004). 35.4% of obese and 7.9% of non-obese subjects had impaired fasting glucose (IFG) (p=0.008). 

Impaired glucose tolerance (IGT) based on 75 gr OGTT was seen in 5.3% of non-obese and 12.5% of obese PCOS women (p=0.314). When we considered HOMA-IR ˃2.30 as cut-off value for existence of insulin resistance based on a recent study in the Iranian population, 72.2% of non-obese and 80.3% of obese subjects had insulin resistance but difference was not statistically significant (p=0.357), but mean of HOMA-IR was higher in obese women and difference between two groups was statistically significant (p=0.004) (10). Among all participants, 41.7% of patients with metabolic syndrome and 12.8% of patients without metabolic syndrome had fatty liver in ultrasound study, showing a significant difference (p=0.006). 

Also, waist circumference in patients with metabolic syndrome (97.00±10.42 cm) was significantly more than subjects without this disorder (77.49±9.34 cm, p<0.001). Results were similar for waist/hip ratio (0.83±0.06 in patients with metabolic syndrome vs. 0.75±0.05 in patients without metabolic syndrome, p<0.001). Comparison of waist circumference in patients with and without fatty liver (96.25±12.3 cm vs. 78.44±10.55 cm) also showed a significant difference (p=0.001). Waist/hip ratio in patients with fatty liver was also significantly morethan subjects without fatty liver (0.81±0.06 vs. 0.76±0.06, p<0.001). There was no statistically significant difference in number of abortions (p=0.786), but complain of mense irregularity was more prevalent in obese PCOS patients (p=0.004). Fourteen patients in obese and 6 in non-obese group suffered from infertility, showing no meaningful difference (p=0.499). Comparison of anthropometeric and biochemical tests in obese and non-obese patients is presented in tables II, III respectively.

**Table I:** Comparison of common complaints in obese and non- obese PCOS patients

**Complaint**	**Non-obese**	**Obese**	**p-value**
Acne	41.5%	40%	0.88
Menstrual irregularity	75.6%	94.3%	0.004
Obesity	0	58.6%	<0.001
Hirsutism	80.5%	88.6%	0.24.2
Hair loss	26.8%	31.4%	0.609
Infertility	14.6%	20%	0.063

**Table II:** Comparison of anthropometric variables in obese and non- obese PCOS patients

**Variable**	**Non-obese**	**Obese**	**p-value**
Age (yr)	22.53 ± 4.46	25.84 ± 5.67	0.001
Weight (kg)	54.59 ± 6.84	77.90 ± 14.39	<0.001
Height (cm)	158.01 ± 6.29	157.20 ± 5.25	0.46
Waist circumference (cm)	70.46 ± 5.09	89.30 ± 10.60	<0.001
Hip circumference (cm)	96.25 ± 5.12	111.38 ± 9.66	<0.001
Waist/hip ratio	0.73 ± 0.04	0.80 ± 0.06	<0.001
Duration of disorder (yr)	7.01 ± 4.79	9.20 ± 5.53	0.029
Ferriman- Galleway score	9.13 ± 6.80	12.34 ± 6.03	0.009
Systolic blood pressure (mmHg)	101.54 ± 11.96	112.49 ± 11.45	<0.001
Diastolic blood pressure (mmHg)	67.07 ± 8.36	74.79 ± 9.53	<0.001
Age of menarche (yr)	13.25 ± 1.37	14.02 ± 7.77	0.536

Data are presented as mean±SD.

**Table III:** Comparison of biochemical tests in obese and non- obese PCOS patients

**Variable **	**Non-obese**	**Obese**	**p-value**
Fasting glucose (mg/dl)	88.24 ± 10.88	95.62 ± 12.88	0.002
Glucose 2 hr after GTT (mg/dl)	98.17 ± 27.01	109.95 ± 30.59	0.045
Fasting insulin (µu/ml)	14.55 ± 9.72	18.54 ± 10.49	0.053
Insulin 2 hr after GTT (µu/ml)	43.05 ± 48.71	66.96 ± 76.87	0.011
HOMA-IR	3.10 ± 1.97	4.46 ± 2.60	0.004
Total cholesterol (mg/dl)	160.90 ± 29.73	180.40 ± 27.90	0.001
Triglyceride (mg/dl)	74.31 ± 31.94	122.80 ± 62.09	<0.001
HDL (mg/dl)	41.83 ± 4.71	42.22 ± 5.88	0.70
LDL (mg/dl)	101.24 ± 21.77	108.60 ± 23.42	0.106
DHEAS (ng/ml)	2898.53 ± 1433.38	2294.00 ± 1129.45	0.018
Total testosterone (ng/dl)	74.40 ± 47.06	78.43 ± 39.47	0.634
Androstenedione (ng/ml)	3.11 ± 1.54	3.00 ± 1.49	0.736
LH/FSH ratio	1.81 ± 1.26	1.58 ± 1.22	0.373
17-OHP (ng/ml)	1.68 ± 0.73	1.62 ± 1.19	0.783
Estradiol (pg/ml)	89.87 ± 57.18	102.03 ± 96.42	0.493
Fatty liver (%)	0	31.3	<0.001
Metabolic syndrome (%)	0	39.4	<0.001
Insulin resistance (HOMA-IR >2.3) (%)	72.2	80.3	0.357

Data are presented as mean±SD.

GTT: Glucose Tolerance Test

HDL: High Density Lipoprotein

LDL: Low Density Lipoprotein

DHEAS: Dehydroepiandrostendine sulfate

LH/FSH ratio: Luteinizing Hormone/ Follicule Stimulating Hormone ratio

HOMA-IR: Homostatic model assessment-estimated insulin resistance

## Discussion

In our obese PCOS patients, symptoms of irregular menses and obesity were more common but there was no difference between obese and non-obese subjects in symptoms of acne, hirsutism, hair loss and infertility. Also no difference in number of abortions and menstrual cycle disorders (as cycles less than 21 or more than 35 days) was seen between two groups. Waist circumference, waist to hip ratio, duration of symptoms, Ferriman-Gallewey score, systolic and diastolic pressure, FBS, glucose and insulin level 2 hr after glucose load, HOMA-IR, total cholesterol and triglyceride levels were all significantly higher in obese PCOS patients. Baldani *et al* showed that obese PCOS patients have higher risk of oligomenorrhea but lower incidence of hirsutism and acne than non-obese subjects. Hyperandrogenemia, insulin resistance, hypercholestrolemia and hypertriglyceridemia were also more common in obese women (11). 

Prevalence of hirsutism and acantosis nigricance was higher in obese PCOS patients in study of Li *et al* (12). Liou *et al* showed that obese PCOS patients have higher risk of oligomenorrhea and hyperandrogenemia but lower risk of acne (13). Menstruation intervals were also longer in obese PCOS women. Siddiqui *et al* reported a statistically significant rise in systolic and diastolic pressures in obese compared to non-obese patients while no significant difference in waist to hip ratio and score of hirsutism was observed between them (14). Use of different criteria for definition of hirsutism is possible cause of differences in various studies. 

Silfen *et al* showed that non-obese cases have higher HDL and lower LDL levels compared to obese PCOS patients. Fasting insulin and fasting glucose to insulin ratio was higher among obese patients (15). Alpanes *et al* stated that adrenal hyperandrogenemia is associated with decreased insulin sensitivity and low total and HDL cholesterol levels (16). Furthermore, LDL and total cholesterol were higher in obese compared to non-obese PCOS subjects in Holte *et al* study (17). Because insulin is one of the major regulators of lipoprotein lipase activity and hyperandrogenemia has an independent role in lipoprotein and lipid metabolism, differences in lipid levels in various studies are somewhat influenced by these factors. In our study no significant difference in total testosterone and androstenedione levels was obtained between obese and non-obese PCOS patients. This finding is consistent with few previous studies (18, 19). 

Moran *et al* showed that basal testosterone in obese cases is significantly more than non-obese PCOS patients whereas the androstenedione level was similar in two groups (20). High testosterone levels correlated with obesity especially the abdominal fat in Wehr *et al* study (21). In our study, serum level of DHEAS was significantly higher in non-obese PCOS cases than obese ones. In general, increased adrenal androgens are seen in 20-50% of PCOS patients (22, 23). 

Moran *et al* showed DHEAS to be significantly higher in non-obese PCOS cases whereas insulin resistance was significantly higher in obese PCOS patients (24). In another study by Moran *et al* PCOS patients with higher adrenal androgens were younger and skinnier and had more hirsutism than PCOS patients with lower adrenal androgens (25). Bernan *et al* study reported an inverse correlation between insulin resistance and DHEAS level (26). Administration of DHEAS supplements to diabetic rodents resulted in improved glucose tolerance and insulin sensitivity (27). 

Also an inverse correlation was observed between DHEAS and insulin resistance in obese women with type 2 diabetes (28). In Chen *et al* study, a positive correlation was found between high level of DHEAS and a better metabolic phenotype, including abdominal obesity, insulin resistance and dyslipidemia (29). Lerchbaum *et al* showed that PCOS women with high DHEAS to free testosterone ratio have a better metabolic profile than PCOS patients with a low ratio (30).

In our study, non-obese PCOS subjects had significantly higher DHEAS levels than obese ones. Waist circumference, waist to hip ratio, systolic and diastolic blood pressures and also fasting glucose and insulin levels, glucose level 2 hr after glucose load, HOMA-IR, total cholesterol and triglyceride levels were all significantly higher in obese PCOS patients compared to non-obese subjects showing a better metabolic profile in our non-obese women. Some studies have shown that as age increases the level of DHEAS and insulin function decrease (31-34). 

Because mean age in our obese PCOS patients was significantly higher than non-obese subjects, lower level of DHEAS and higher insulin resistance in obese PCOS women was to some extent due to higher mean age in our obese group. No case of fatty liver based on ultrasound study and metabolic syndrome was diagnosed in our non-obese PCOS patients whereas the frequency of fatty liver and metabolic syndrome was 31.3% and 39.4% in obese PCOS patients, respectively. Moreover, the prevalence of fatty liver and also waist circumference and waist/hip ratio was higher in patients with metabolic syndrome. The waist circumference and waist/hip ratio values were also significantly more in patients with fatty liver than patients without this condition. 

These data show that higher waist circumference and waist/hip ratio which means more abdominal obesity is associated with the occurrence of fatty liver and metabolic syndrome. Gambarin *et al* studied 88 PCOS patients and showed that 55% of them had liver steatosis whereas 39% of patients with fatty liver did not fulfill the obesity criteria (35). There was a significant positive correlation between BMI and HOMA-IR with liver steatosis and patients with liver steatosis had lower HDL level and higher prevalence of impaired fasting glucose, impaired glucose tolerance and diabetes. There was a significant difference in the prevalence of steatosis in PCOS patients and controls in Vassilatou *et al* study (36.8% vs. 20%, respectively) (36). 0All PCOS patients and controls with metabolic syndrome had liver steatosis. Bohdanohicz *et al* reported fatty liver in 57.6% of PCOS women and 49.6% of controls based on ultrasound studies (37). Women with PCOS and liver steatosis had greater waist circumference, waist/hip ratio and BMI than PCOS women without liver steatosis. In Dokras *et al* study, the prevalence of metabolic syndrome was 47.3% in the PCOS group and 4.3% among controls and the risk of metabolic syndrome in all age-groups was higher in PCOS women rather than controls (38).

However, no difference in androgen levels in PCOS patients with and without metabolic syndrome was reported. They concluded that the prevalence of metabolic syndrome in PCOS subjects is 11 times more than age-matched controls. Park *et al* showed a 3.5 times higher prevalence of metabolic syndrome in PCOS women compared to the urban women population in Korea (39). In Li *et al* study, insulin resistance was seen in 43.23% of all PCOS patients (40). 82.86% of cases with insulin resistance were in the obese group (BMI >25) and 20.49% were in the lean group (BMI <25). 

In addition, a positive correlation between BMI and waist/hip ratio with HOMA-IR was found. In our study the prevalence of insulin resistance was 72.2% in non-obese and 80.3% in obese PCOS patients. HOMA-IR was higher in obese PCOS subjects indicating significant difference between obese and non-obese PCOS patients (p=0.004). Our limitation in this study was unequality in sample sizes in two groups due to refer of more obese patients to our clinic. Increasing the duration of study for resolving this problem was not successful. Therefore, we finally enrolled 45 non-obese and 70 obese PCOS patients in this study. This limitation must be considered in interpretation of results. 

## Conclusion

Metabolic consequences of obesity and insulin resistance are more prevalent in obese PCOS patients than non-obese ones, however, an increase in adrenal axis activity and DHEAS level is significantly more prevalent in non- obese patients. Therefore, the metabolic aspects of PCOS must be considered in such patients especially in obese subjects. 
